# Method of Building Detection in Optical Remote Sensing Images Based on SegFormer

**DOI:** 10.3390/s23031258

**Published:** 2023-01-21

**Authors:** Meilin Li, Jie Rui, Songkun Yang, Zhi Liu, Liqiu Ren, Li Ma, Qing Li, Xu Su, Xibing Zuo

**Affiliations:** 1 Department of Geographic Information, Information Engineering University, Wutong Street High-Tech District, Zhengzhou 450001, China; 2 School of Computer Science & Technology, Beijing Institute of Technology, Haidian District, Beijing 100081, China

**Keywords:** machine vision, remote sensing images, building detection, SegFormer, atrous spatial pyramid

## Abstract

An appropriate detection network is required to extract building information in remote sensing images and to relieve the issue of poor detection effects resulting from the deficiency of detailed features. Firstly, we embed a transposed convolution sampling module fusing multiple normalization activation layers in the decoder based on the SegFormer network. This step alleviates the issue of missing feature semantics by adding holes and fillings, cascading multiple normalizations and activation layers to hold back over-fitting regularization expression and guarantee steady feature parameter classification. Secondly, the atrous spatial pyramid pooling decoding module is fused to explore multi-scale contextual information and to overcome issues such as the loss of detailed information on local buildings and the lack of long-distance information. Ablation experiments and comparison experiments are performed on the remote sensing image AISD, MBD, and WHU dataset. The robustness and validity of the improved mechanism are demonstrated by control groups of ablation experiments. In comparative experiments with the HRnet, PSPNet, U-Net, DeepLabv3+ networks, and the original detection algorithm, the mIoU of the AISD, the MBD, and the WHU dataset is enhanced by 17.68%, 30.44%, and 15.26%, respectively. The results of the experiments show that the method of this paper is superior to comparative methods such as U-Net. Furthermore, it is better for integrity detection of building edges and reduces the number of missing and false detections.

## 1. Introduction

A building is a key artificial ground object with a roof and walls and is the central place of human habitation. Building information is essential to carry out various urban and regional activities such as city planning [1], disaster monitoring [2], traffic management [3], and scientific planning of the eco-environment [4]. Accurate extraction of building information from remote sensing images is difficult due to the complexity of building features and surrounding ground objects such as buildings, vegetation, roads, and bare areas. Building features can be diverse due to differences in the material and configuration of ground objects [5]. A great deal of unnecessary, non-building information can be confounded in the detection network when the spatial distribution of surface features is complex, resulting in poor classification detection effects. Traditional image processing with manual selection of design features is time-consuming and labor intensive. Consequently, it is difficult to satisfy real-time latest requirements and to apply on a large scale. At present, there is a large gap between intelligent judgment and drawing technology research and its practical applications.

In recent years, research on the extraction of building information has been conducted using a high-resolution remote sensing image classification method based on the convolutional neural network (CNN) [6]. These studies reviewed the semantic representation capacity of the neural network for road network extraction, building detection, and crop classification [7]. The feature extraction module presented by a fully convolutional network (FCN) is widely used for the semantic segmentation of buildings [8]. The U-Net network proposes the idea of skip connections based on the encoder-decoder. These skip connections in the U-Net network are used to fuse deep and shallow features to elevate the precision of semantic segmentation. The U-Net network can extract more intact features compared with the FCN network [9,10]. By cascading multi-scale-resolution features, a high-resolution network (HRNet) can effectively decrease the feature information loss, make the extracted multi-scale features more abundant, and result in the pixel-level extraction of building information [11,12]. The pyramid scene parsing network (PSPNet) aggregates the multi-scale features of distinct areas through the pyramid pooling module and the pyramid scene parsing module, thereby elevating the integrity of building edges [13]. A DeepLab series network can acquire a preferable edge perception effect by introducing depthwise separable convolution aggregating multi-scale information features [14,15]. The SegFormer network can ameliorate the extracted invalid non-building features to a certain degree by cutting off the positional encoding and utilizing multi-layer perception (MLP) for feature extraction [16]. Although the above-mentioned feature extraction networks have high detection precision, the issue related to information loss has not been adequately ameliorated. Additionally, these detection networks have not focused on extracting building features in a complex environment and suppressing the effect of the extracted non-building information. Consequently, the extraction of building features in an intricate environment needs to be further strengthened.

In existing research, the convolutional neural network model has been directly applied to the remote sensing field [17]. The high-resolution remote sensing building extraction method based on deep learning has both advantages, as evidenced by multiple experiments, as well as some problems. For one thing, the network model specially constructed for the detection or classification of buildings in remote sensing images is relatively lacking [18]. For another, it is necessary to investigate more efficient ways to acquire semantic information and detail features of buildings because buildings on remote sensing images are influenced by factors such as data acquisition, different scales, imaging conditions, intricacy terrain, building shadows, and building inclination.

The objective of this study is to address the poor applicability of the existing models on building extraction from remote sensing images. This study presents a novel approach for building detection in remote sensing images based on the SegFormer network structure and thereby optimizes and improves the above issues. In this study, the transposed convolutional network [19] is coupled with the sampling module to correlate semantic feature information better. This coupling results in the efficacious screening of feature categories and reinforces the construction of network models for building features. Issues such as loss of building semantic and detail features and missing and false detections are addressed by using strongly supervised datasets. The atrous spatial pyramid pooling [20] decoding module is fused to capture lucid building edge information to address the above-mentioned issues resulting from factors such as building inclination and shadow shading.

## 2. The Building Extraction Network of Improved SegFormer

### 2.1. SegFormer

SegFormer [21], a semantic segmentation network, was developed by combining Transformer with visual classification and was co-launched by the Nvidia Corporation and the University of Hong Kong in 2021. It has the following improvements in addition to the potential of high processing efficiency and dense prediction of Transformer series.

① A lightweight decoder is constructed, and MLP is issued for feature aggregation [22].
② The hierarchical structure design is adopted. The multi-layer feature map is attained via the hierarchical Transformer Encoder [23] without positional encoding. The high-resolution shallow features and low-resolution fine features are obtained at multiple scales.

SegFormer stresses robustness and validity in Transformer series networks. While having challenges against image interference, the semantic segmentation task is completed efficiently and with higher precision. Issues of building detection in remote sensing images can be solved better due to the improvements made based on SegFormer.

### 2.2. Improvement Strategy

**Figure 1:**
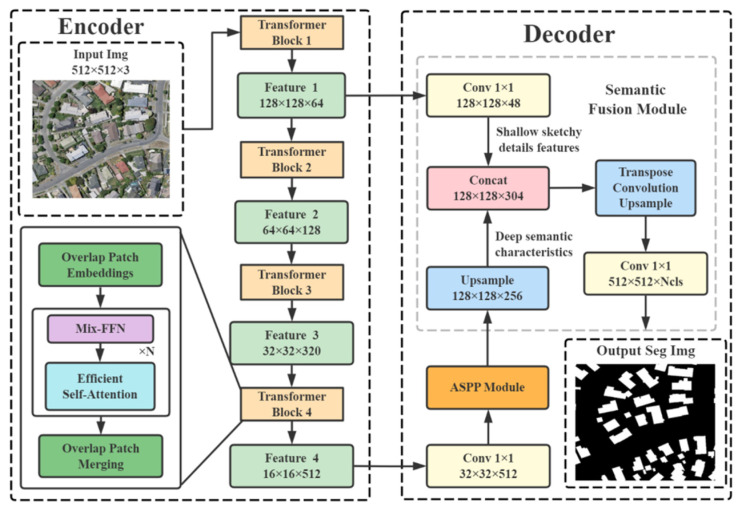
Schematic diagram of the network structure.

The improved network structure presented in this paper is shown in Figure 1. The improvement has two main aspects:

(1) The structure of the detection network is optimized, and the sampling of semantic and detailed features of buildings is reinforced to address the poor applicability of the existing models for building extraction from remote sensing images. The transposed convolutional network is coupled with the sampling module to correlate semantic feature information, fuse high and low-layer feature information, and cascade inter-layer receptive fields. This can lead to receding the impact of spatial heterogeneity and intricate ground object background and intensifying the establishment of a detection network model for building features. Multiple normalization activation layers are fused [24]. The screening efficiency of building semantic information is enhanced by reducing the quantity of the parameters, inhibiting the disappearance of the gradient, and speeding up the convergence. To efficiently gain the semantic information features for buildings, the effective screening and classification of the intra-class feature information are realized by embedding the activation layer [25].
(2) The decoding output part of the model is optimized, and the atrous spatial pyramid pooling (ASPP) decoding module is used to address issues such as excessive interference, loss of semantic and detailed feature information of buildings, and missing and false detection of building features extracted from remote sensing images. Multi-scale contextual information is explored by applying multi-sampling rate atrous convolution [26] and multi-receptive field convolution on the input feature map. A sufficient amount of contextual information is captured by pooling operations at different resolutions, and lucid target boundaries are captured by gradually recovering spatial information. The semantic fusion module is used in the output part to aggregate rough information in the shallow layer and fine information in the deep layer. The interaction of feature information from different layers is achieved by using the shuffle [27]. The shuffle is applied to address issues such as the partial loss of building information and the lack of long-distance information. It is also helped to efficaciously relieve the loss of feature information resulting from the difference in image resolution due to miscellaneous data sources. Consequently, issues such as the loss of edge details of multi-scale buildings and the missing and false classification of tiny buildings can be addressed.

#### 2.2.1. Sampling Module Based on the Transposed Convolutional Network

The information of remote sensing images is intricate, with many interferences. The higher the distinction degree between detected ground objects and background information, the easier the identification of stable features with strong anti-interference. Therefore, in this paper, we design the enhancement extraction mechanism for interlayer features. This is based on transposed convolution from the angle of reinforcing the expression of enhancing features and inhibiting futile background information. The learning is intensified in the training iteration to acquire the optimum sampling parameters by establishing an adaptive learning model.

##### Transposed Convolution

The size of the feature map gradually lessens following the convolutional operation of the images several times. Transposed convolution can efficaciously couple the feature information of the high layer and the low layer, and cascade the receptive fields between layers. This will address the issue of over-flow of non-building information due to inconsistent image resolution or due to different scales in the feature map.

In convolution operation C, the convolution Y output for the n-dimensional vector X can be expressed as CX = Y, and its transposed convolution can be expressed as X = CTY. The purpose of transposed convolution is to add holes and fillings to restore more semantic information via rearranging the input and kernel. The specific implementation pattern of transposed convolution is as follows:

By overlooking the number of channels, it is hypothesized that the stride is s and the padding is p. The shape of the input tensor is nh×nw, the shape of the convolution kernel is kh × kw, and the transposed operation yields nh × nw intermediate results. Each intermediate result is a tensor of (n_h_ + k_h_ − 1) × (n_w_ + k_w_ − 1) (The parameter is initialized to 0). The approach to calculating the intermediate tensor is as follows: Each element in the input tensor is multiplied by the convolution kernel; thereby, the tensor of k_h_ × k_w_ is attained, which replaces a part of the intermediate tensor. The position of the replaced part of each intermediate tensor corresponds to the position of the element in the input tensor. In the end, all intermediate results are added to obtain the final result. Computational Formula (1) of the intermediate tensor is as follows

**Figure 2:**
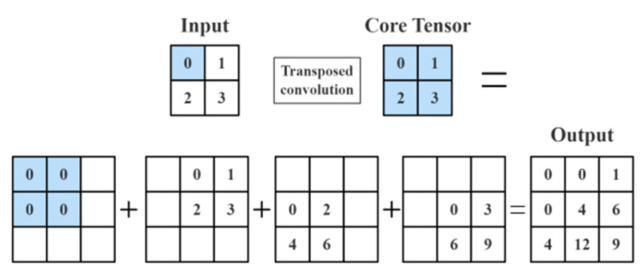
The example of the sampling method (when n and k are 2, s = 1, p = 0).

**Figure 3:**
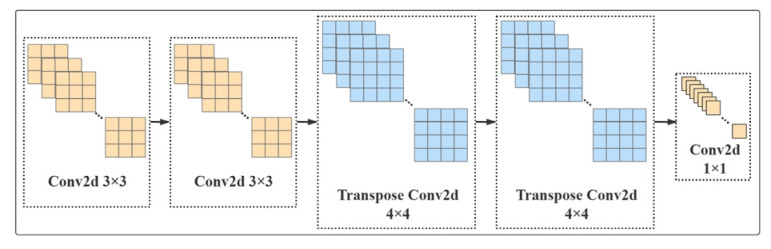
Structure diagram of the transposed convolutional network: yellow is convolution, blue is transpose convolution.

(1) Y[i:(i+h),j:(j+w)]+=X[i,j]×K

when n and k are 2, s = 1, p = 0, and the input parameters are {0, 1, 2, 3}; the calculation form is shown in Figure 2:

Based on the aforesaid theory, the transposed convolutional network designed in this paper is shown in Figure 3: Multi-layer perception output channels of 4C, two 3 × 3 2D convolution layers, two 4 × 4 2D transposed convolution layers and one 1 × 1 2D convolution layer are embedded in the subsampled part by following the characteristic map. Through the transposed convolution operation, some information can be efficaciously recovered while the fuzzy boundary is retained, thereby outputting more targeted detailed information and semantic information features.

##### Multiple Normalization Activation Layers

The convolution operation abstracts the local features of the building images; however, it neglects the interrelationship between pixels. This can lead to inter-class inconsistency, dramatically affecting the reliability and integrity of building edge segmentation. The addition of the extra normalization and activation layers upon convolution operation can result in a stable selection of feature parameters by holding back over-fitting regularization expression and simultaneously averting the influence of outliers and extremes. Furthermore, the addition of both layers can inhibit noise interference, strengthen the expression capacity of the network, and can increase the focus on feature channels and spatial positions with strong significance and enormous information. As a result, the internal correlation of images or features can be acquired easily; thereby, the issue of precise extraction of inter-class differences of buildings can be solved.

**Figure 4:**
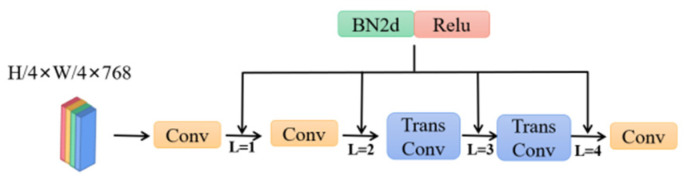
Schematic diagram of the inserting position of the normalization activation layer: red is activation function; red is activation function; green is batch normalization; yellow is convolution; blue is transpose convolution.

The normalization layer is set based on the introduction of the transposed convolutional network, and the setting mode is shown in Figure 4.

#### 2.2.2. Spatial Pyramid Pooling Decoding Module Fused Atrous Convolution

The fusion approach of absolute skip connection of semantically dissimilar features is unable to address the semantic gap resulting from the lack of multi-scale features in the design of the network. As a consequence, the extraction capacity of the network will be severely restrained to large-scale building edges and tiny building objects. Based on this, this paper designs a spatial pyramid pooling decoding module fused with the atrous convolution. Firstly, the deep-layer network features are dilated according to distinct dilation rates, the contextual information of multi-scale features is captured by the atrous spatial pyramid pooling module, and the global average pooling module is introduced to supplement, thereby refining the contextual information. Secondly, in light of the semantic-guided fusion module, the corresponding relationship between feature pixels at different layers is established, and the deep-layer semantic information is fused into the shallow image details in a bottom-up way. The input feature maps are dilated and divided into groups according to the dilation coefficient, the features at different scales are extracted by adopting distinct dilation kernels, and the shuffle of extraction features is conducted to achieve information about the interaction of different layers and to strengthen global semantic information expression.

##### Atrous Convolution (Dilated Convolution)

In contrast to the traditional convolution layer and pooling layer, the atrous convolution possesses the following advantages: ① After the replacement of the atrous convolution, the training of reasonably dilated new parameters can be avoided when the calculation parameters are increased, and the performance can be continuously optimized in the training process. ② The feature maps at different resolutions can be attained by setting different atrous rates to alter the size of the receptive field, which decreases the loss of position information in the subsampled process.

**Figure 5:**
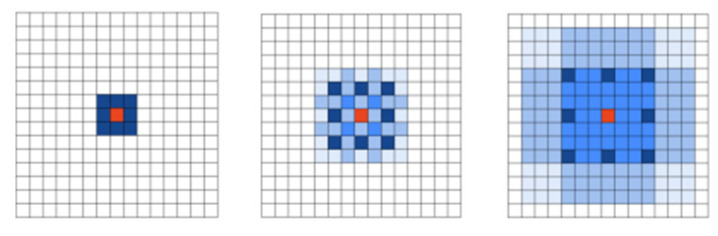
Atrous convolution with reasonable dilation rate.

**Figure 6:**
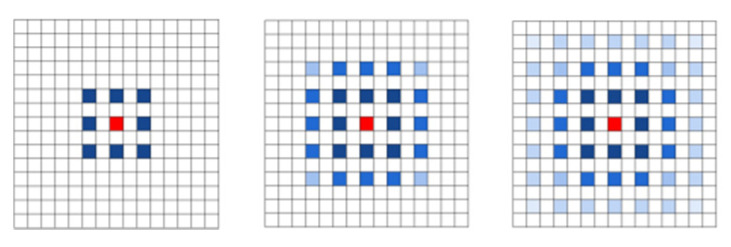
“Gridding Effect” of atrous convolution.

In this paper, the conventional convolution is replaced by the atrous convolution, and the receptive field in the pooling process is altered by changing the atrous rate of the atrous convolution. The experiment discovers that distinct dilation rates have a certain influence on the performance of the model as well. The atrous convolution mode with a reasonable dilation rate is shown in Figure 5. The authors of [28] show that: ① the use of atrous convolution with the identical dilation rate can result in the discontinuity of the convolution kernel, leading to the “Gridding Effect”, as shown in Figure 6; ② the dilation rate combinations cannot contain the common divisor greater than 1, and the dilation rate Ri must satisfy Formula (2) as follows; ③ it is hypothesized that the dilation rates corresponding to n atrous convolutions with the convolution kernel size of k × k are [r1,…, ri,…rn], respectively, and R2 ≤ k should be satisfied, where ri represents the dilation rate of the ith atrous convolution, Ri represents the largest dilation rate of the ith atrous convolution, and the default is Rn = rn. Based on the above conclusion, this paper designs the atrous convolution combination with the dilation rate of 1, 3, 11, 17

(2) $$R_{i}=\max\left[R_{i+1}-2r_{i},R_{i+1}-2\left(R_{i+1}-r_{i}\right),r_{i}\right]$$

##### Atrous Spatial Pyramid Pooling

The atrous spatial pyramid pooling module consists of a series of atrous convolutions and spatial pyramid pooling structures with different dilation rates. Multi-scale information of the image is extracted through the parallel connection of multiple atrous convolutions with distinct dilation rates. The global information of the image is gained by introducing global average pooling (GAP). When using single atrous convolution, the ASPP module can overcome the disadvantages of local information loss and long-distance information deficiency due to the Gridding Effect. The feature information of different scales and clear high-resolution building edge information can be acquired without using a pooling layer.

**Figure 7:**
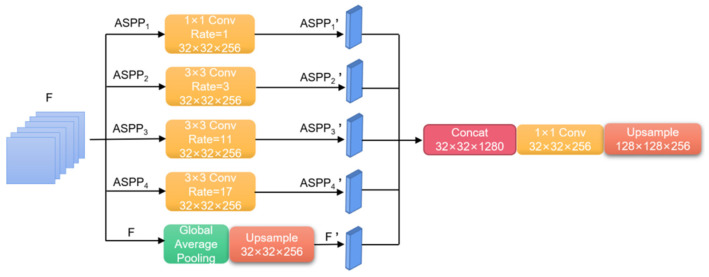
Schematic diagram of the ASPP module.

Firstly, the ASPP module dilates the input feature F into i groups, which are denoted as ASPPi (i = 1, 2, 3, 4) (Figure 7). Input calculations of different groups can alter the dense connection mode between the original feature channels and can gain the gridding parameters of different corresponding positions. Secondly, the multi-scale object context information of each grouping feature of ASPPi is attained by conducting the F pooling, upsampling, and then fusing with the corresponding feature ASPPi’.

##### Semantic-Guided Fusion Module

**Figure 8:**
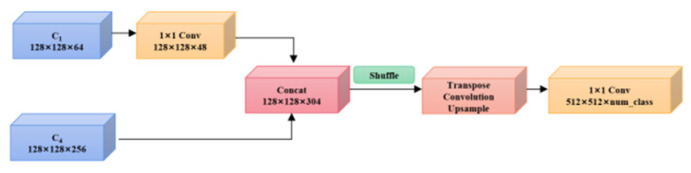
Semantic fusion module.

**Figure 9:**
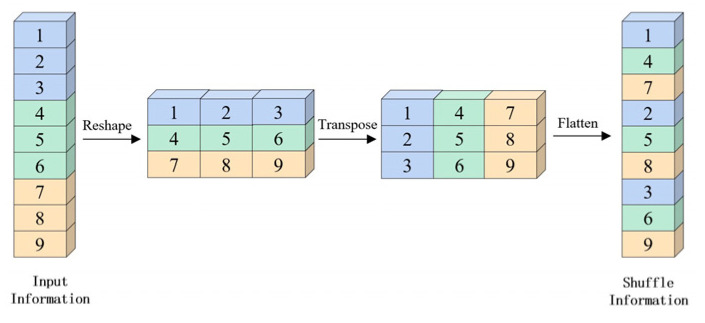
Shuffle.

This paper puts forward an idea of using the Semantic Fusion Module (SFM) (Figure 8). In this module, the detailed information obtained by rough feature extraction in the shallow layer is fused with fine semantic information in the deep layer gained by the ASPP multi-scale atrous pyramid pooling network operation. Then, the channel shuffle of fusion information is conducted (Figure 9). The feature channel sequence of the original grouping is disarranged and rearranged by “remodeling-transposition-remodeling.” The “semantic flow” information between features with different resolutions is forecasted by network autonomous learning. Rough features are rectified to fine features with higher resolution, and pixels between features with different resolutions are “aligned.” The feature layer with abundant multi-scale information is obtained by shuffling information from different channels, reconciling multi-scale information, and intensifying the information exchange between feature channels of different groupings. This will effectively transmit semantic information from the deep layer to the shallow layer and the efficacious fusion of features with different resolutions.

## 3. Experiment and Analysis

### 3.1. Experimental Dataset

The effectiveness and generalization of the method presented in this paper are validated by the multi-method tests. The results are compared with the Aerial Image Segmentation Dataset (AISD) [29], the Massachusetts Buildings Dataset (MBD) [30], and the Wuhan University (WHU) aerial remote sensing image dataset [31].

(1) The AISD contains four ground object labels—buildings, roads, water systems, and vegetation. In this paper, building labels are only used for the analysis. The dataset covers areas of Berlin, Chicago, Paris, Potsdam, and Zurich, and the images have a resolution of 0.3 m. This dataset includes 1672 images with 3328 × 2816 pixels deriving from Google Earth and pixel-level label of Open Street Map.
(2) The MBD covers the cities and suburbs areas of Boston and contains 151 images with 1500 × 1500 pixels. The occupied area of each image is 2.25 km², with a resolution of 1 m.
(3) The WHU includes 204 images with 512 × 512 pixels in Satellite Dataset(Global Cities), and the coverage area is the global urban satellite images, with a resolution of 0.3 m.

**Figure 10:**
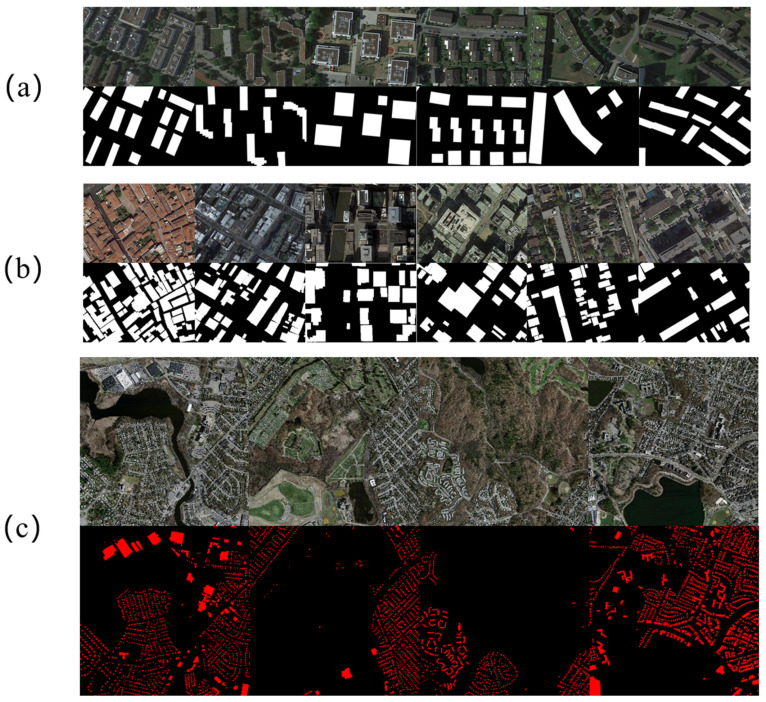
Samples of (**a**) the AISD, (**b**) the WHU dataset, and (**c**) the MBD.

Samples of different datasets have been shown in Figure 10.

The division of datasets is shown in Table 1:

### 3.2. Experimental Environment and Parameter Setting

The experimental operating system is Windows 11, the CPU version is 12th Gen Intel(R) Core(T.M.)i9-12900H, the GPU is NVIDIA GeForceRTX3080TI, and the deep learning framework is torch 1.11.0 + cu115. The experiment adopts the transfer learning strategy, and the pre-training weights in the ImageNet-1K dataset are used for training. The accuracy of the detection results is enhanced by the network model by carrying out transfer learning and by depending on its capacity of unsaturated continuous learning. The training process is divided into the freezing stage and the unfreezing stage. The training configuration information is shown in Table 2:

### 3.3. Ablation Experiment

Ablation experiments are performed on the AISD, the MBD, and the WHU dataset with SegFormer as the baseline system to validate the improved mechanism presented in this paper. The settings of experimental parameters are consistent with the experimental environment, and the results are shown in Table 1.

#### 3.3.1. Ablation Experiment with Different Mechanism Modules

To validate the improved mechanism, the ablation experiments are conducted on the AISD, the MBD, and the WHU dataset, and SegFormer is used as the baseline system. The promotion effect of model performance is assessed through the evaluation indicators of mean Intersection over Union (mIoU), F1 score, precision, and recall. Therein, mIoU is referred to the percentage mean of intersection-over-union of predicted building pixels and actual building pixels; the F1 score is taken into account the influence of precision and recall; precision is meant by the percentage of pixels of the building correctly predicted by the model to the actual building; recall is referred to the percentage of the correctly predicted building to the actual building. Suppose that the predicted building is represented as T, the non-building as F, the actual building as P, and the non-actual building as N, then Formulas (3)–(6) of the four evaluation indicators are as follows. The network training process is visualized, and a comprehensive analysis of the effect of the improved network is conducted.

(3) $$\mathrm{mIoU}=\frac{1}{k+1}\overset{k}{\underset{i=0}{\sum }}\frac{\mathrm{TP}}{\mathrm{FN}+\mathrm{FP}+\mathrm{TP}},\left(k=1\right)$$

(4) $$\mathrm{Precision}=\frac{\mathrm{TP}}{\mathrm{TP}+\mathrm{FP}}$$

(5) $$\mathrm{Recall}=\frac{\mathrm{TP}}{\mathrm{TP}+\mathrm{FN}}$$

(6) $$F1=2\times \frac{\mathrm{Precision}\times \mathrm{Recall}}{\mathrm{Precision}+\mathrm{Recall}}$$

(1) Ablation experiments with different improved mechanisms

The original network model and the network model with different mechanisms are tested on three datasets, and the comparison of the evaluation indicators is shown in Table 3.

When the experiments are conducted on the AISD, the MBD, and the WHU dataset, the precision evaluation indicators obtained for the improved network are superior compared to those obtained for the original network (Table 3). This suggests the validity of the improved network for building extraction suggested in this paper. For the improved network, the enhancing effects of the precision indicators on the MBD are the best, where mIoU, F1, precision, and recall indicators increased by 9.76%, 10.54%, 10.58%, and 10.45%, respectively. However, the applicability of the original network to this dataset is inferior, with a mIoU of 66.09%. After the optimization, the improved network shows a stronger capability of feature extraction and fusion. This can improve the focus and recovery of semantic and feature information of different layers and efficaciously elevate the segmentation precision of the whole model.

(2) Visualization of the training process

**Figure 11:**
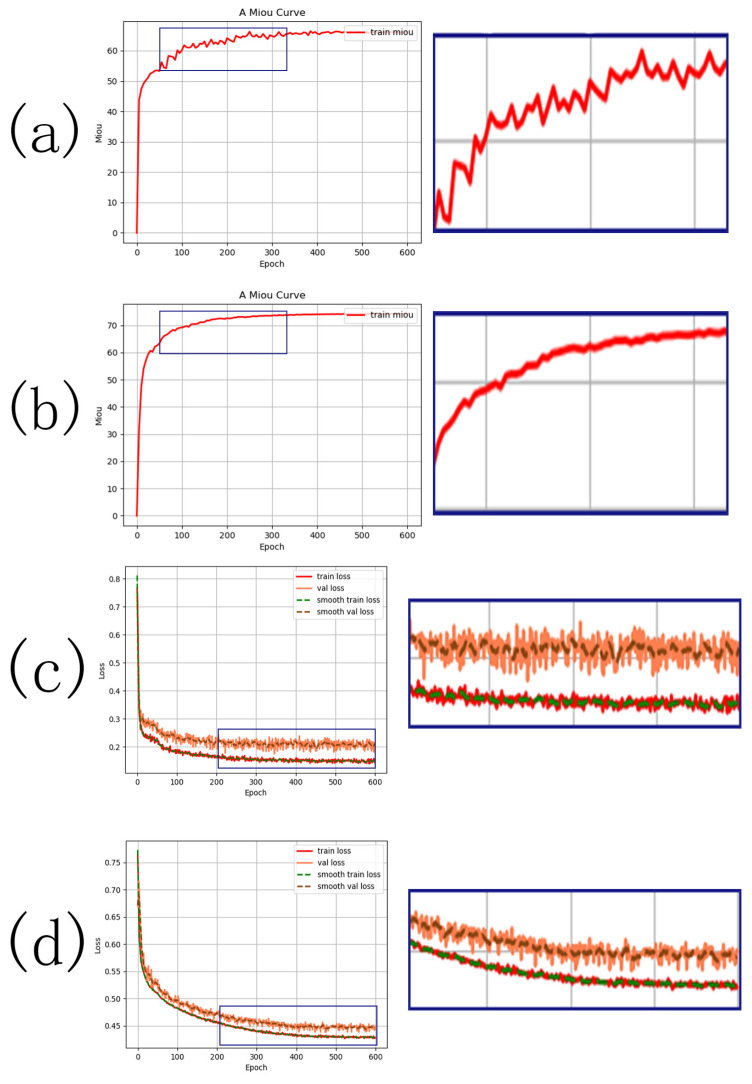
(**a**) Variation curve of training precision of the original model. (**b**) Variation curve of training precision of the improved model. (**c**) Variation curve of training loss of the original model. (**d**) Variation curve of training loss of the improved model.

During the training process, variations of the loss value and segmentation precision of both original and improved SegFormer network models are tested on the MBD and are shown in Figure 11.

During the training process, the overall training curve shows a gentle tendency, and the fluctuation of the precision curve of the improved network is smaller than that of the original network after adding the transposed convolutional network module (Figure 11a,b). Higher precision of the improved network can be realized in a shorter time compared with the original network. During the training process, the improved network can dynamically adjust according to the contextual information, as shown by the contrast of the dynamic variation curves of precision and loss. Furthermore, the improved network can more efficiently supplement the detail features and semantic features, possessing preferable extraction precision, generalization capacity, and universality.

#### 3.3.2. Ablation Experiment of Multiple Normalization Activation Layers

The multiple accumulations on the MBD are adopted to analyze the improvement effect of the stacking normalization activation layer. This will increase the understanding of the availability and contribution of the added modules in the established transposed convolutional networks, and investigate the promotion effect of cascading multiple normalization activation on the network expression capacity and precision. The addition method is shown in Figure 4.

The selected evaluation indicators of mIoU, F1, precision, and recall are improved along with the multiple superpositions of normalization activation layers (Table 4). Among them, mIoU, F1, precision, and recall increased by 3.11%, 1.27%, 0.58%, and 1.81%, respectively. The precision improvement is reached to the maximum when the four normalization activation layers are embedded in the convolution network. Experiments validate that the detection capacity of the improved network can be enhanced after the introduction of multiple normalization activation layers.

#### 3.3.3. Dilation Rate Ablation Experiment of Atrous Spatial Pyramid Pooling Decoding Module

This paper also verifies the effects of different dilation rate combinations on the performance of the ASPP module. The comparative experimental results based on the MBD are shown in Table 5, which demonstrates that the effect of non-identical continuous convolution is superior. The precision of the combination of 1, 3, 6, 9 is increased by 0.33% compared with the combination of 1, 2, 2, 2, and the effect of prime number combination is better with the large dilated kernel. When the combination is 1, 5, 11, 17, the precision is elevated by 0.27% compared with the combination of 1, 6, 12, 18. The second dilation rate is not greater than the maximum size of the convolution kernel. However, when the combination is 1, 3, 11, 17, the precision is enhanced by 2.15% relative to the combination of 1, 5, 11, 17. Based on this, the discontinuous atrous convolution combination of 1, 3, 11, 17 with the common divisor of 1 and a large dilation rate is chosen. During the experiment, the precision of this combination is enhanced by 1.96–3.93% relative to other combinations. The loss of relevant information can be reduced, and the effect of capturing contextual information of objects at different scales can be advanced at a reasonable dilation rate.

### 3.4. Comparison Experiment

The experimental dataset is obtained from the AISD, the MBD, and the WHU dataset, and the analysis is based on high-precision pixel-level labels. Therefore, the scope, distribution, and geometric outline of buildings on images can be precisely shown. In this experiment, the label information of the original dataset is visualized on images and compared with the experimental results. The validity of this improved algorithm is demonstrated using several kinds of classical mainstream deep learning semantic segmentation algorithms. High-resolution net (HRnet), PSPNet, U-Net, DeepLabv3+, and the original SegFormer network are selected to compare the algorithm of this paper, and comparative results are presented in Table 6:

The overall experimental precision of the improved method is superior to that of the original SegFormer model and the other mainstream methods used in the last few years (Table 6). Therein, on the AISD, mIoU, F1, precision, and recall are up to 88.43%, 93.81%, 93.85%, and 93.77%, respectively; on the MBD, these four indicators are 75.85%, 88.24%, 93.07%, and 83.89%, respectively; on the WHU dataset, the values of corresponding four indicators are reached to 81.69%, 91.09%, 92.46%, and 89.76%, respectively. On three datasets, relative to the comparison method, the optimal promotion of mIoU of the improved method is 17.68%, 30.44%, and 15.26%, and the optimal promotion in precision is 11.17%, 20.02%, and 11.86%.

When diverse extraction methods are used to test the MBD, the experimental precision of all algorithms is significantly lower than that of the other two datasets (Table 6). This is because all images in the MBD are large-scale scene images. As shown in Figure 10, the coverage area of a single image is extremely wide, and there is a significant difference between the image scale of the MBD and that of the other two datasets. Additionally, when extracting buildings in wide-range scenes, the building styles, spectral features, and shadow features of the MBD are more diverse and complicated than those of the other two datasets. The experimental effect of the segmentation network on the MBD is not ideal. Nevertheless, after the network optimization, the improved network has efficaciously enhanced the detection precision of the MBD, among which mIoU, F1, precision, and recall have increased by 9.76%, 10.54%, 10.58%, and 10.45%, respectively. After the network improvement, the experimental detection precision has been elevated by the order of magnitude. This is indicated by the strengthened applicability of large-scale scene images, better performance on large-scale datasets, and stronger robustness in the face of intricate buildings and variable environments, validating the effectiveness of the improved algorithmsection.

The model complexity and efficiency of Hrnet, PSPNet, UNet, Deeplabv3+ and the original SegFormer network are compared with the algorithm in this paper, and the results are shown in Table 7. In the experiment, the input size is set to 512 × 512 when calculating GFLOPs. The training time is the time required for one iteration of the MBD. As can be seen from Table 7, the mIoU of the algorithm in this paper improves greatly when GFLOPs, parameters and training time increase slightly, which is of high application value while costing less.

**Figure 12:**
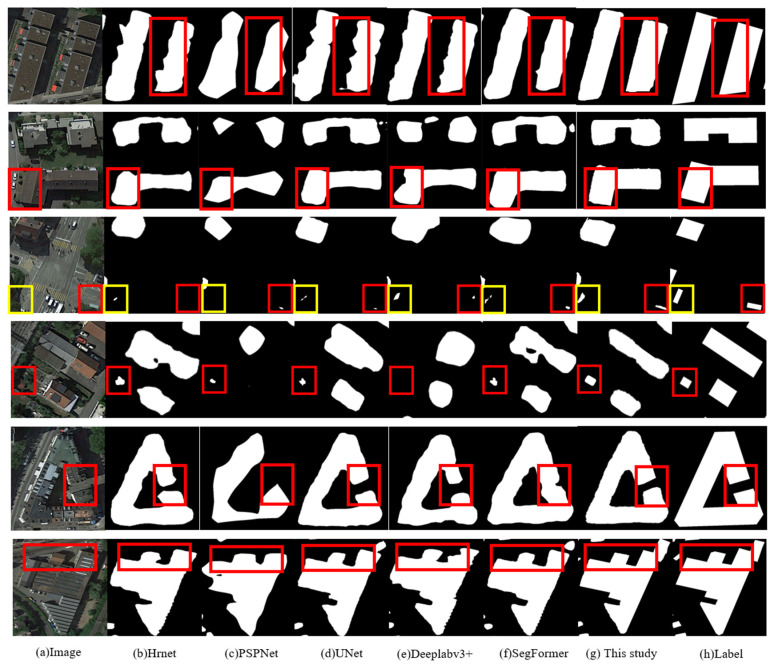
Detection effects of different networks: (**a**) original image; (**b**) Hrnet; (**c**) PSPNet; (**d**) UNet; (**e**) Deeplabv3+; (**f**) SegFormer; (**g**) this study; (**h**) label.

Figure 12 shows the experimental prediction results of HRnet, PSPNet, U-Net, DeepLabv3+, the original SegFormer algorithm, and the algorithm presented in this paper. The performance of various algorithms is assessed according to the visualization of detection results. Therein, column (a) is the original image, column (h) is the dataset label, columns (b)–(f) are the detection results of the other networks, and column (g) is the detection results of the improved network. According to Figure 12, while testing on the identical image, the detection results of the improved network are better than those of other networks in the following aspects:

(1) The integrity of the detection edge is better, and the boundary transition is more regular and smoother: the architectural form of the building is neat. The 1st and 2nd rows in Figure 12 show that the detection of a single independent building by the HRnet, PSPNet, and SegFormer is poor, especially depicting the right-angle side of the building. The U-Net and improved network can exactly locate the sideline of the building. Nevertheless, in edge detection, the improved network is more intact and reasonable in the transition of edge detection details than the U-Net network. The improved network in this study can better differentiate the background from the building boundary relative to the SegFormer network. The improved network revises the regular edges of buildings better and obtains apparent enhancement in the detection effect.
(2) The construction of building feature information is improved, the missing and false detection due to the inter-class similarity of ground objects is efficaciously inhibited, the multi-scale generalization capacity is strong, and the detection rate of tiny buildings is enhanced. In the 3rd and 4th rows of Figure 12, when the color of tiny buildings in the original image is similar to that of the ground objects in surrounding background and when there is a large-scale difference between tiny buildings and the surrounding buildings, the phenomenon of missing buildings occurs in the PSPNet and DeepLabv3+ experiments. In the experiments of HRnet, U-Net, and SegFormer, though the positioning of some buildings can be realized, they cannot be validly detected. In contrast, the improved network exactly locates tiny buildings and detects their distribution range and edges.
(3) The reservation of detailed feature information is abundant and intact, and the differentiation capacity for buildings and their surrounding backgrounds is powerful. According to the 5th and 6th rows in Figure 12, when detecting polygonal buildings and aiming at the transition of edge texture, the evident edge fitting, the severe loss of edge details in angle and corner, and the erroneous judgment of building distribution on a large scale exist in the comparison algorithms. However, the improved network restores more intact details compared with other networks. There is little difference in the distribution range and outline when the detection results of the improved network are compared with the label information.
(4) Limited by feature similarity: the spectral similarity interferes greatly and the inhibition of negative features is weak. When the background with similar characteristics is mixed, the building classification performance is mediocre. There are still great challenges in the discrimination of detection algorithms.

## 4. Conclusions

To obtain high-precision extraction of buildings in remote sensing images, based on SegFormer, this paper presents a method for building detection in remote sensing images. This novel method consists of an improved sampling mechanism and a decoding structure, which ameliorates issues such as missing and false detection of buildings, poor edge integrity, and poor effects of intelligent interpretation of remote sensing images resulting from inter-class similarity and intra-class inconsistency of buildings. The main improvements of the new algorithm are the use of a spatial pyramid pooling decoding module fusing atrous convolution and the introduction of the transposed convolutional network to optimize the sampling mode. These improvements make the algorithm efficiently screen positive information about buildings while inhibiting and filtering negative information and easing the issue of missing semantic information and detail features. The ablation and contrast experiments are carried out on the remote sensing image AISD, MBD, and WHU dataset. The experimental results show that the improved algorithm is more accurate in boundary segmentation than the other methods, and the false detection of buildings is reduced. It can be applied to large-scale scene detection and intelligent judgment and drawing, which provides strong support for surveying and mapping production. In the next step, we will explore the building detection method to suppress the spectral imaging interference of remote sensing images.

## Figures and Tables

**Table 1:** Datasets used in this paper.

Dataset	Category	Number	Resolution
AISD	train	1504	2611 × 2453
	val	168	2611 × 2453
	test	168	2611 × 2453
MBD	train	135	1500 × 1500
	val	16	1500 × 1500
	test	16	1500 × 1500
WHU	train	183	512 × 512
	val	21	512 × 512
	test	21	512 × 512

**Table 2:** Training configuration of the dataset.

Item	AISD	MBD	WHU
Input size	2611 × 2453	1500 × 1500	512 × 512
Train size	512 × 512	512 × 512	512 × 512
Test size	512 × 512	512 × 512	512 × 512
Freeze epoch	50	50	50
UnFreeze epoch	500	600	200
Batch size	8	16	4
Optimizer	Sgd	Sgd	Sgd
Learning decay	Cos	Cos	Cos
Weight decay	0.0005	0.0005	0.0005
Learning rate	1.00 × 10^−5^	1.00 × 10^−5^	1.00 × 10^−5^

**Table 3:** Comparison of experimental precision between the improved network and the original network.

Dataset	Network	Evaluation Indicator			
		mIoU	F1	Precision	Recall
AISD	segf	82.17	90.11	89.93	90.3
	segf + Transconv	83.58	90.97	90.9	91.05
	segf + ASPP	83.19	91.3	91.8	90.8
	This study	**88.43↑6.26**	**93.81↑3.7**	**93.85↑3.92**	**93.77↑3.47**
MBD	segf	66.09	77.70	82.49	73.44
	segf + Transconv	74.23	85.44	87.35	83.61
	segf + ASPP	72.74	86.97	92.49	82.08
	This study	**75.85↑9.76**	**88.24↑10.54**	**93.07↑10.58**	**83.89↑10.45**
WHU	segf	79.5	88.23	88.17	88.31
	segf + Transconv	80.69	89.01	88.86	89.18
	segf + ASPP	81.23	91.16	92.95	89.45
	This study	**81.69↑2.19**	**91.09↑2.86**	**92.46↑4.78**	**89.76↑1.45**

**Table 4:** Ablation experiment of the normalization activation layer.

The number of normalization activation layers	L = 1	×	✓	✓	✓	✓	Optimal mechanism promotion
	L = 2	×	×	✓	✓	✓	
	L = 3	×	×	×	✓	✓	
	L = 4	×	×	×	×	✓	
mIoU		72.74	74.02	74.78	75.42	**75.85**	3.11
F1		86.97	87.02	87.52	87.54	**88.24**	1.27
Precision		92.49	92.5	92.74	**93.11**	93.07	0.58
Recall		82.08	82.16	82.87	82.6	**83.89**	1.81

**Table 5:** Effects of different dilation Rates on the performance of the ASPP module.

Dilation Rate Combination	mIoU
1, 2, 2, 2	71.92
1, 3, 6, 9	72.25
1, 2, 5, 11	72.96
1, 6, 12, 18	73.43
1, 5, 11, 17	73.7
1, 3, 11, 17	75.85

**Table 6:** Precision comparison of different methods.

Detection Network		HRnet	PSPNet	U-Net	DeepLabv3+	SegFormer	Methods of This Paper	Optimal Promotion (%)
Dataset	Evaluation Indicator (%)							
AISD	mIoU	77.17	70.75	80.39	76.37	82.17	**88.43**	17.68
	F1	86.95	82.56	89.00	86.42	90.11	**93.81**	11.25
	Precision	86.74	82.68	88.94	86.26	89.93	**93.85**	11.17
	Recall	87.17	82.45	89.07	86.59	90.30	**93.77**	11.32
MBD	mIoU	55.93	45.41	73.58	53.52	66.09	**75.85**	30.44
	F1	68.71	60.50	83.68	67.64	77.70	**88.24**	27.74
	Precision	76.10	73.05	86.92	78.15	82.49	**93.07**	20.02
	Recall	62.62	51.63	80.67	59.62	73.44	**83.89**	32.26
WHU	mIoU	75.32	66.43	77.10	72.22	79.50	**81.69**	15.26
	F1	85.41	78.87	86.67	83.21	88.24	**91.09**	12.22
	Precision	85.62	80.60	87.84	84.14	88.17	**92.46**	11.86
	Recall	85.20	77.22	85.54	82.30	88.31	**89.76**	12.54

**Table 7:** Network complexity analysis.

Network	GFLOPS /G	Parameters /M	Computation Time (sec/epoch)	mIoU
HRnet	79.539	29.538	14	75.32
PSPNet	5.873	2.377	7	66.43
U-Net	451.94	24.891	20	77.1
DeepLabv3+	52.846	5.818	7	72.22
SegFormer	13.674	3.72	15	79.5
Methods of this paper	27.17	3.903	18	**81.69**

## Data Availability

The data used to support the findings of this study are included in this article.
